# A Systematic Approach of the Intrauterine Morphogenesis of the Human Palpebral Apparatus

**DOI:** 10.1080/15476278.2022.2066453

**Published:** 2022-05-22

**Authors:** Octavian Munteanu, Florin-Mihail Filipoiu, Monica Mihaela Cirstoiu, Roxana Elena Bohiltea, Tiberiu Augustin Georgescu, Adrian Dumitru, Andra-Ioana Băloiu, Mihai-Alin Publik, Ioan-Andrei Petrescu

**Affiliations:** a Department of Anatomy, Carol Davila University of Medicine and Pharmacy, Bucharest, Romania; b Department of Obstetrics and Gynecology, Carol Davila University of Medicine and Pharmacy, Bucharest, Romania; c Department of Pathology, Carol Davila University of Medicine and Pharmacy, Bucharest, Romania; d Faculty of Medicine, Carol Davila University of Medicine and Pharmacy, Bucharest, Romania

**Keywords:** embryology, eyelid, palpebral apparatus, development, anatomy, morphogenesis

## Abstract

The human eyelid embodies a vast diversity of functions. Acting as a protective shield for the ocular apparatus and as a light regulator in the sight process, eyelids stand a fascinating – yet omitted – role in facial aesthetics, serving as a racial trait by which humankind succeeded to manifest heterogeneity as a species. These assumptions are precisely forecasted right from in-utero life through intricate processes of growth and cell differentiation. In the Department of Anatomy of “Carol Davila” University of Medicine and Pharmacy, we performed morphological assessments on 41 embryos and fetuses with gestational ages ranging from 6 to 29 weeks. This study aims to illustrate the morphogenesis of eyelids in human embryos and fetuses and highlight macroscopic features which could potentially have significant clinical implications in ophthalmic pathology.

## Introduction

The palpebral apparatus consists of a complex ensemble of structures that are assigned to achieve specific purposes. The eyelids act as a protective cover for the ocular globe and serve as a mechanical lubricator along with the lachrymal apparatus.^1,2^ Although they lack in thickness, the eyelids also perform an important role as they filter and block light in the process of accommodation.^3^

### Relevant anatomy

The upper and bottom eyelids resemble each other in terms of the stratification of their multi-layered setup. Allegedly, specialists divide the eyelid into 3 lamellae: anterior, middle, posterior.^1,2,4,5^ The anterior lamella is represented by the skin and the Orbicularis Oculi Muscle (OO). The palpebral part of OO tightens the eye closure.^2,4^ The middle lamella refers to the Orbital Septum (OS), which is inserted at the level of Aditus Orbitae (Aditus Orbitalis, also known as the opening of the orbit).^6^ It separates the pretarsal structures from the underlying ones and acts as a secondary insertion point for Levator Palpebrae Superioris Muscle (LPS).^4,7,8^ The posterior lamella is composed of the Tarsal Plates (TP), LPS muscle, and Conjunctiva.^1,2^ TPs are 2 dense fibrous tissue masses that have a rather structural role, maintaining the lids in a steady position.^4,8^ Superior Tarsus is also attached to the Müllerian Muscle, or Müller’s Muscle, a smooth muscle strip deeply embedded between LPSs’ striate fibers, which contributes to the Sympathetic Tonus of the eyelid.^1,2,5,9^

***The embryology*** of eyelids has fairly become a matter of concern to the extent of which both molecular biology and micro-architectural findings were illustrated in the literature. In this regard, eyelid formation entails an elaborate instrumentation between epithelial and mesenchymal growth^10–12^ along with the differentiation process of embryonic tissue into specific components. Epithelial and glandular derivatives originate in the surface ectoderm while mesenchymal structures develop from neural crest cells.^10,13^ The mesoderm gives rise to striate muscle fibers in the region.^10^

There are up to 5 phases in the eyelid morphogenesis mentioned in previous research: formation, fusion, development, separation, and maturation.^10, 14–16^ The findings show that eyelids first appear as eyelid folds in either week 6^10^ or week 7^17,18^ of gestation, through a mesenchymal proliferation surrounding the optic placode.^18–20^ By the end of week 8, the folds fuse with the medium of the periderm cell layer,^18^ allowing both palpebral and orbital structures to form and develop. Specialists demonstrated that eyelid fusion is essential in the differentiation process for the ocular adnexa, preserving structures from the amniotic fluid and its renal excretion metabolites.^10,16^ The OO muscle appears around week 9, TP primordium becomes apparent around week 11 together with lash follicle anlagen, meibomian gland anlagen, and Orbital Septum primordium.^10,16–18^ By week 14 the lid components display a layered arrangement and continue to develop until week 20 when eyelid separation starts. From this on, TP, OS, glands, fatty tissue, and muscles reshape to their definite anatomy to provide proper appearance and functionality at birth.^10,17,18^

All these events were described in extenso due to the important role that signaling pathways play. Extracellular molecules such as FGF10 (Fibroblast Growth Factor 10), TGF-α (Transforming growth factor-alpha), Activin B, and HB-EGF (Heparin-binding EGF-like growth factor) modulate signaling cascades involved in cell migration and differentiation processes.^19,21^ Moreover, disruptions of such pathways along with other genetic dysfunctions lead to multi-systemic disorders in which eyelid anatomy is compromised.^19,22–24^

Despite being of interest to many researchers, many of the eyelid development studies cover the subject on mouse specimens, and human studies are rather scarce. Additionally, there is little information about macroscopic findings and their correlation to clinical implications. Due to the fact that the plastic and oculofacial surgery is constantly developing and the knowledge in the pediatric ophthalmic field is expected to become more satisfactory, this study aims to clarify and simplify how eyelid and orbital structures progress and develop relations in fetal life, focusing on the external view of the eyelid through developmental stages.

## Materials and methods

We have performed dissections on human embryos and fetuses from the specimen collection of the Anatomy and Embryology Department, College of Medicine, Carol Davila University of Medicine and Pharmacy. The specimens range in gestational age between 6 and 29 weeks, established by crown-rump length measurements according to Carnegie staging system and were grouped into 3 evolutionary stages: Stage A – 1 to 8 weeks (embryo); Stage B – 9 to 20 weeks and Stage C – 21 to 29 weeks. All embryos and fetuses were previously fixed in a 10% neutral buffered formalin solution. We have analyzed the palpebral apparatus of 41 human embryos and fetuses. The ones which presented mechanical damage in the orbit area due to either obstetrical procedure mishappenings or maneuvering circumstances were excluded from further analysis. Therefore, 79 palpebral apparatuses have been evaluated. Additionally, an extensive microscopical assay has been performed for 21 specimens. They were treated for histological slides, cut into 3-µm-thick sections along the frontal and transversal planes, and stained with routine hematoxylin-eosin coloration. We have analyzed the evolution of the eyelids and reviewed the correlation between the eyelid differentiation process and the development of the ocular globe and its adnexa. We compared the macroscopic findings to microscopic sections of the same gestational age.

The procedures we used and the tests we conducted comply with the Helsinki Declaration of 1975, as amended in 2000, as well as in the national legislation. We gathered written consent from all patients who consented to have their aborted fetuses utilized in the study. The Morphological Science Department of the “Carol Davila” University of Medicine and Pharmacy has given its permission to the study.

## Results

### Stage A

**Figure 1.**
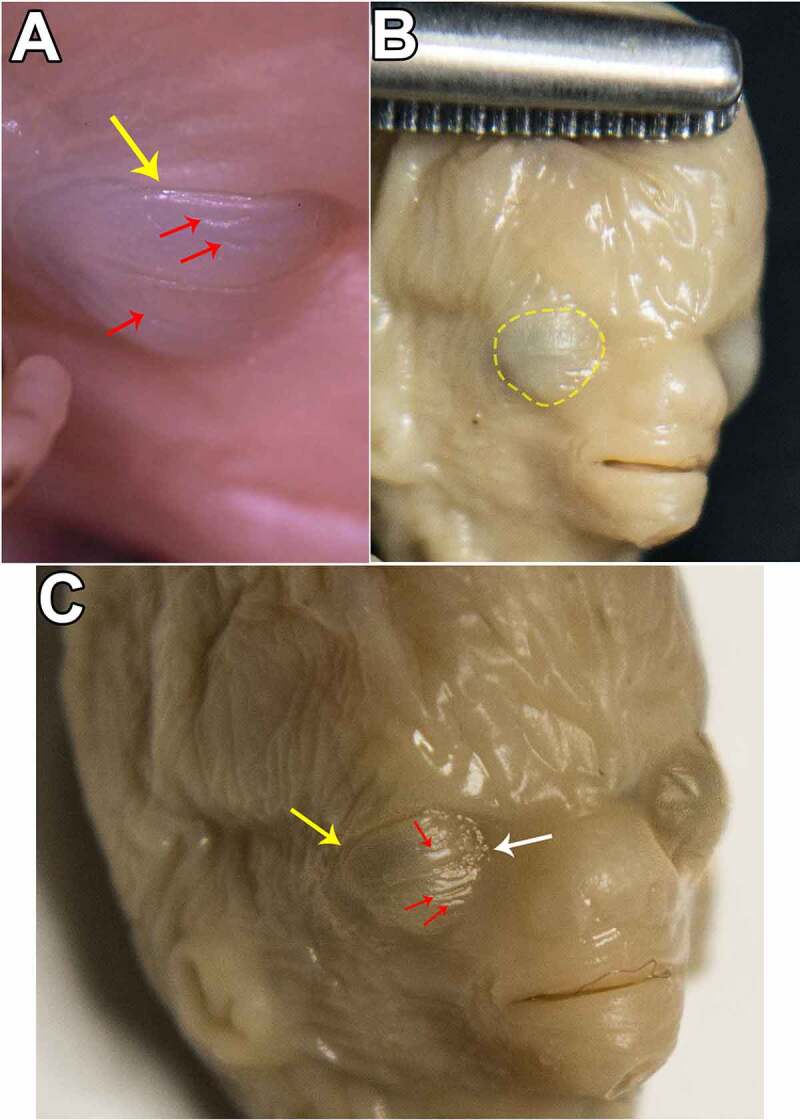
STAGE A; eyelid of a 6-week old embryo, oblic-anterior view; picture a was taken using a dissection microscope. yellow arrows and yellow dashed contour highlight the groove circumscribing aditus orbitae. white arrow points toward the transverse groove marked by the palpebral fusion. red arrows indicate the creases on both upper and lower eyelids which give the aspect of a wrinkled eye.

**Figure 2.**
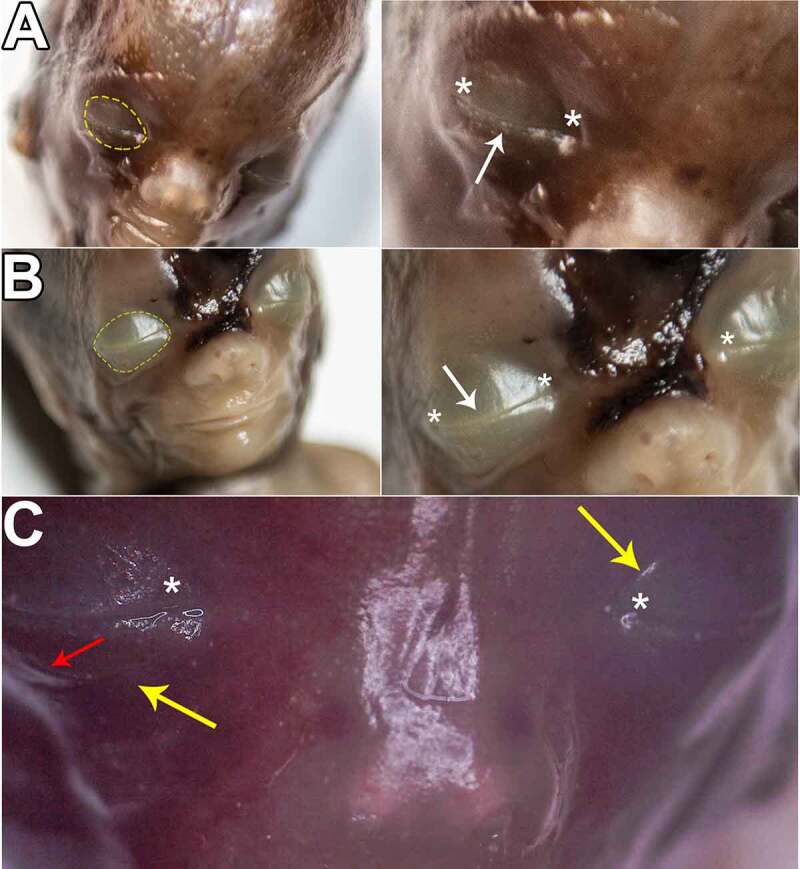
STAGE A; Eyelids of: A, C – 7-week-6-day old embryo and B – 8-week-3-day old embryo; picture C was captured with the dissection microscope. yellow arrows and yellow dashed contour – groove corresponding to aditus orbitae. white arrows – palpebral fusion; notice that both medial and lateral limit, respectively the medial and lateral palpebral commissures (white asterisks) are very alike at this gestational age. red arrows – transversal creases along the upper and lower eyelids; notice they appear less often than in younger specimens. White asterisks – palpebral commissures.

Although at this stage macroscopic elements are quite limited in number, they are easy to identify and describe. Thus, tardily in this stage, the eyelids display a fused aspect, where a fine transversal lineament can be observed. This equatorial contour alongside the future canthi (palpebral commissures) serves as a border between the newly fused eyelids. Moreover, there was no noticeable difference between lateral and nasal canthi. It is very important to mention that, because in the meantime face development is still in progress and orbital bones are not completely developed either, the position of the eyes is toward lateral and thus, a rather exophthalmic aspect is more often observed. Consequently, the area corresponding to Aditus Orbitae is circumscribed by an integumentary circular groove. We consider this to be an external indicator for the bone insertion point of the Orbital Septum. This exophthalmic aspect is apparent until early Stage B, and as the embryo evolves, eyelid width changes. It is initially thin and displays fine transversal creases on both upper and lower eyelid, and by the time glandular, muscular, follicular anlagens become more evident in microscopy, eyelids turn into a thick, smooth/stretched layer above the globe. (Figure 1,2)

### Stage B – microscopic analysis

**Figure 3.**
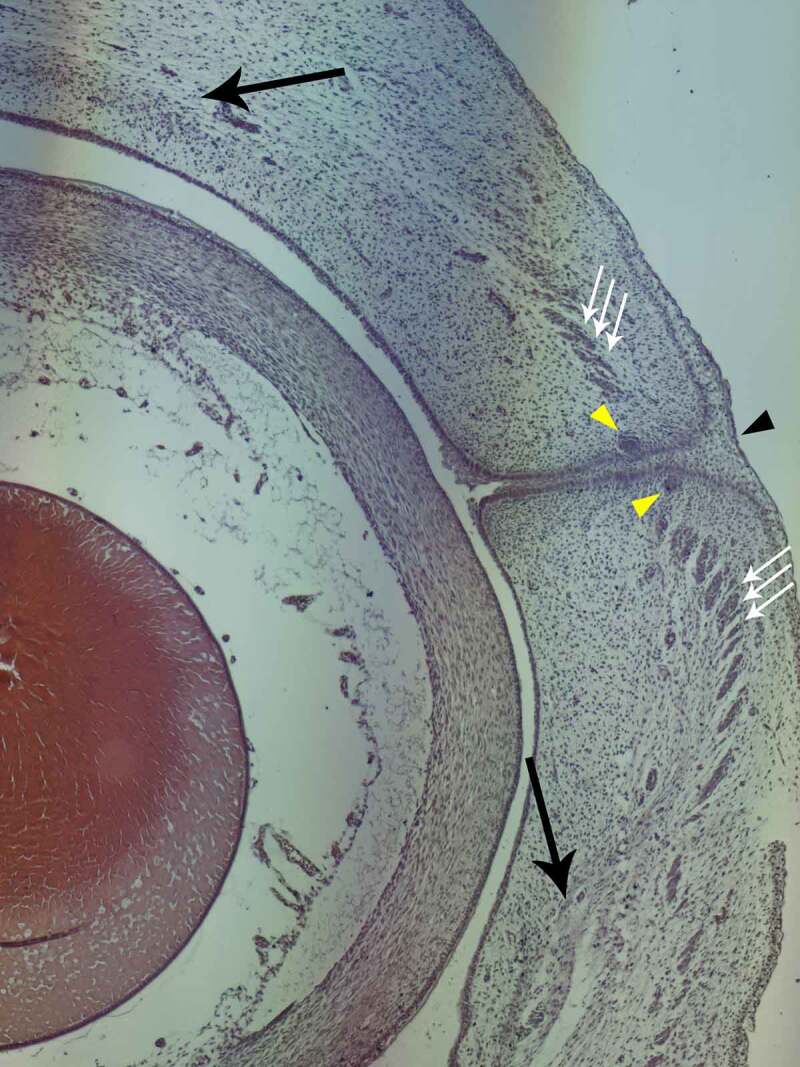
STAGE B; frontal microscopic section of the eye of a 10-week old fetus; hematoxylin-eosin staining; magnification 5x. black pointer – periderm cell proliferation at the fusion level of the eyelids. yellow pointers – eyelash follicle anlagens. black arrows – TP primordium appearing as a mesenchymal condensation. Triple white arrows – OO muscle primordium.

As we previously stated, the exophthalmic aspect continues to be visible during the first 2–3 weeks of this stage. Due to this, we were able to analyze the microscopic morphology in frontal sections. Thus, besides a well-defined cellular layer that serves as a seal between the upper and lower eyelids, OO, LPS, TP, and hair follicle primordia can be observed as tissue condensations. Extraocular Muscles are observable as well, respecting classic anatomy, along with the Optic Nerve and the Lacrimal Gland Primordium. (Figure 3).

### Stage B – macroscopic analysis

**Figure 4.**
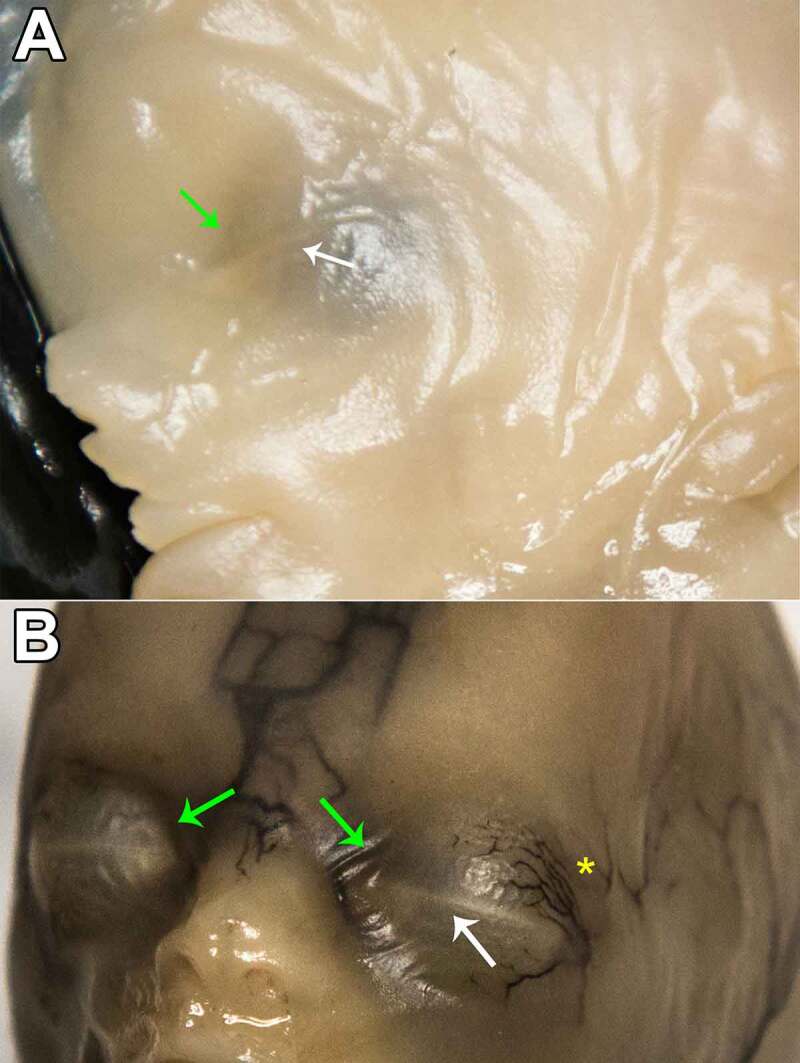
STAGE B; A: 11–12-week old fetus and B: 14-week old fetus. white arrow – palpebral fusion. yellow asterisk – superficial vessels. green arrows – superior palpebral sulcus primordium; as the eyelids develop, the groove circumscribing aditus orbitae fades and is no longer noticeable; however, an invagination remains visible above the upper palpebral commissure, which will eventually become the superior palpebral sulcus.

**Figure 5.**
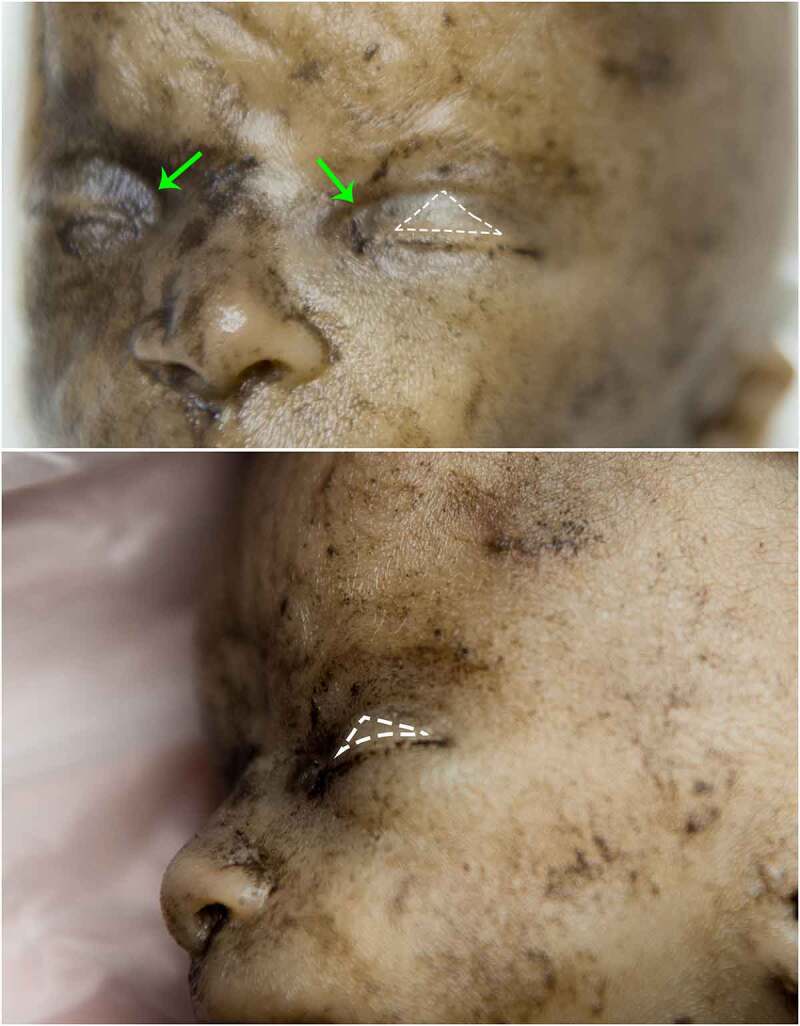
STAGE B 18-week old fetus, oblic-frontal view (up) and lateral view (down). white dashed contour – tarsal plate protruding at the eyelid surface in a triangular shape, with its base oriented inferiorly. green arrows – superior palpebral sulcus; note that the invagination described in Figure 4 is now more prominent toward medial and fully elongated above the upper eyelid.

The Palpebral Fusion becomes more apparent during this stage, as it changes its aspect. It can initially be seen rather as a protruding transversal line than an equatorial groove. Soon after, the fusion develops again as an invagination between the two eyelids, as they grow as well. Superficial vessels were observed. (Figure 4) Late during stage B, Superior Palpebral Sulcus becomes apparent as a skin recess that defines medially the Medial Palpebral Fissure. Superior TP projects the overlying skin in a triangular shape whose base corresponds to the Palpebral Fusion and with its vertex oriented toward superior. (Figure 5).

**Figure 6.**
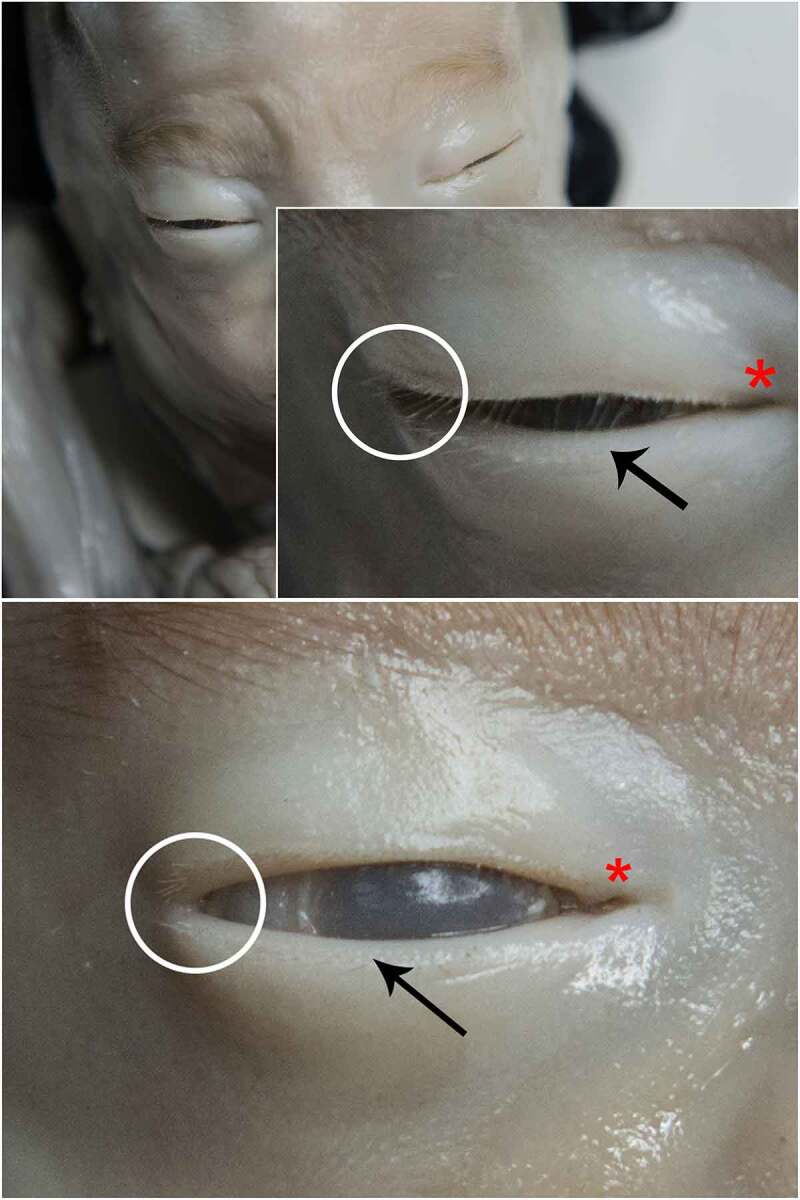
STAGE B; 20-week old fetus. Eyelids are separated at this gestational age. Lateral and medial palpebral commissures appear different in aspect. the caruncle is observable at the level of the medial canthus (red asterisk). white circles focus on the eyelashes. black arrows – wet line; no lacrimal punctus is yet noticeable at the medial end of the inferior wet line.

By the end of this stage, eyelids appear to be separated and eyelashes become noticeable. If the eyes are open, a rudimentary wet line can be seen behind the eyelash emergence line. Yet, no lacrimal punctus is visible during this time. Additionally, eyes are closer to the median line, no exophthalmic aspect is visible anymore and eyelids get a more definite appearance, together with palpebral fissures, canthal angles, and caruncle. (Figure 6).

### Stage C

**Figure 7.**
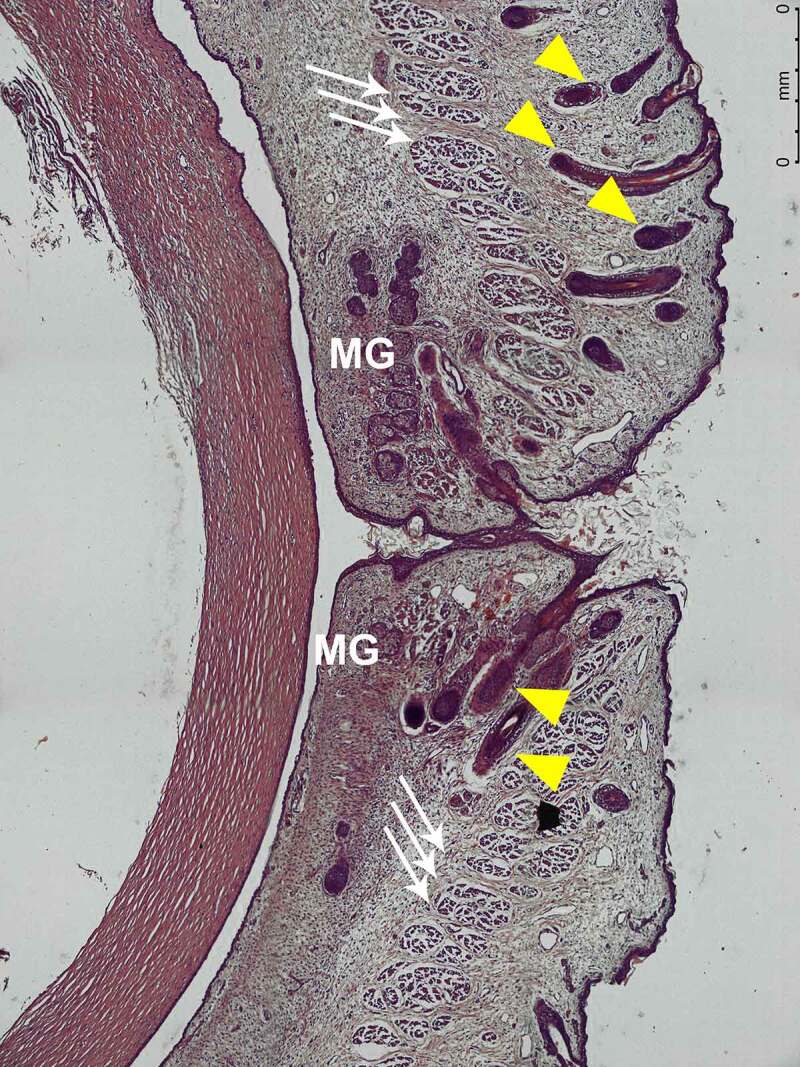
STAGE C; sagittal macroscopic section of the eye of a 25-week old fetus; hematoxylin-eosin staining; magnification 5x.Yellow pointers – eyelash follicles. Tripple white arrows – OO muscle. MG – Meibomian Gland.

**Figure 8.**
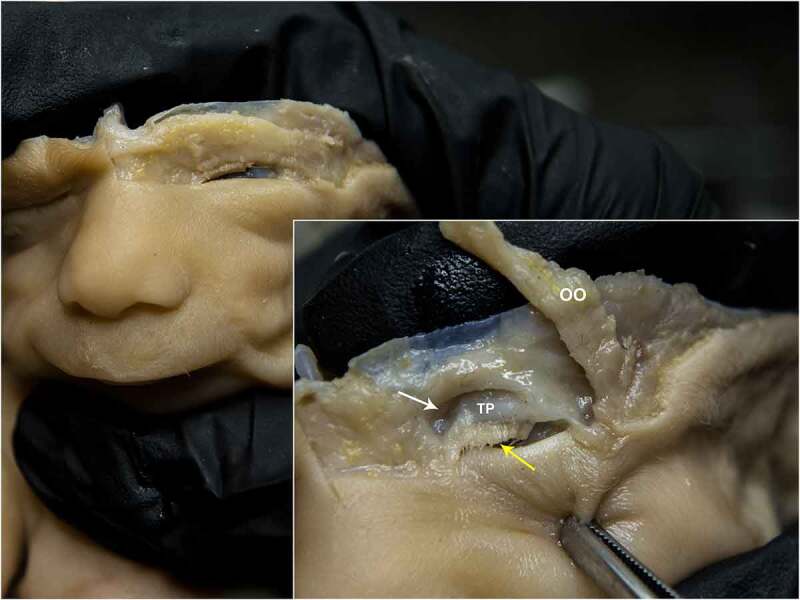
STAGE C; dissection of the upper eyelid of a 24-week old fetus; OO – orbicularis oculi muscle. white arrow – orbital septum inserting on aditus orbitae. TP – tarsal plate laying underneath the OS. Yellow arrow – eyelash follicles. Skin and OO were removed to highlight the middle lamina; TP is visible through the transparency of the OS.

**Figure 9.**
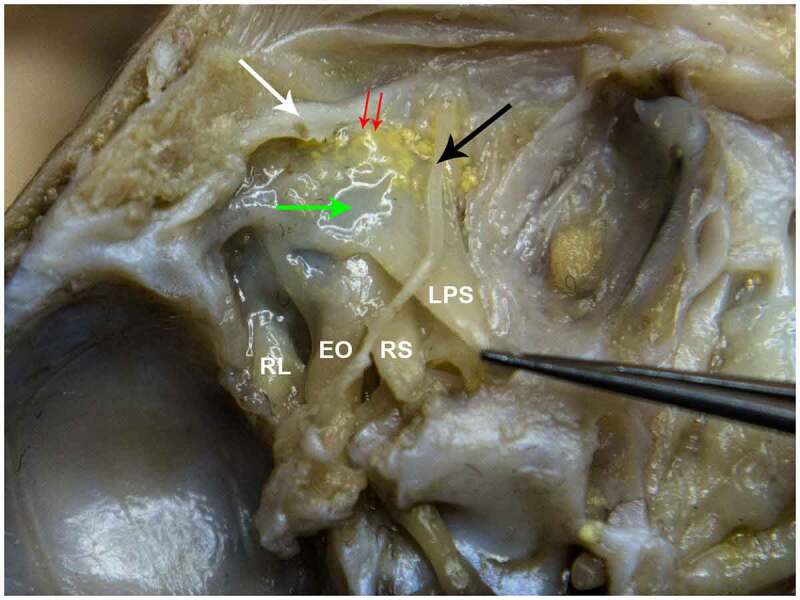
STAGE C; Dissection through the superior wall of the left orbit of a 24-week old fetus. white arrow – orbital septum. LPS – levator palpebrae superioris muscle. RS – Rectus Superior Muscle. EO – External Oblique Muscle. RL – Rectus Lateralis Muscle. Black Arrow – Supraorbital Nerve. Green arrow – tendon of the LPS inserting on the TP. Double red arrow – Post Septal Fat.

**Figure 10.**
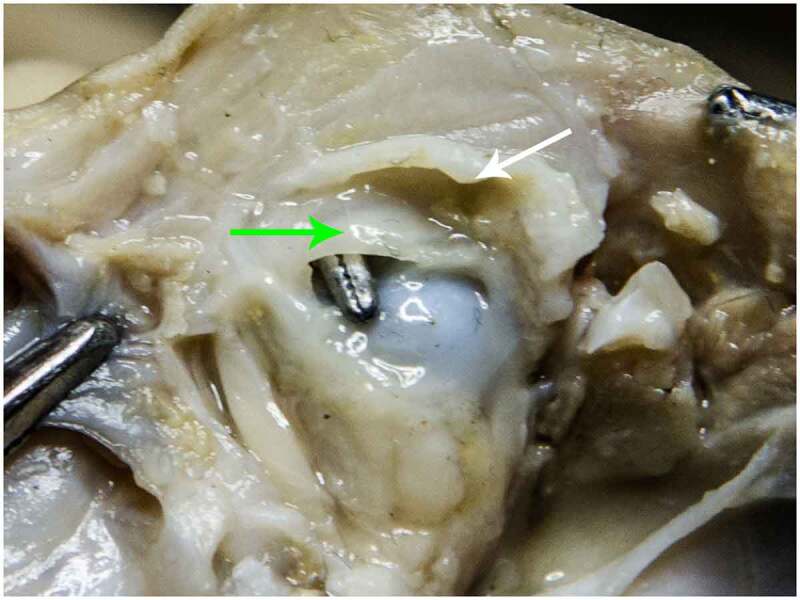
STAGE C; Dissection through the superior wall of the right orbit of a 24-week fetus; LPS muscle was removed. White arrow – Orbital Septum; the post septal fat was removed. Green arrow – LPS Tendon; the tendon was preserved to highlight its insertion to the superior TP, laying right above the tip of the forceps sitting on the ocular globe.

By the time fetuses reach such gestational age and until parturition, the overall view does not suffer major changes. Eyes and eyelids respect the typical anatomy of the area. The OO muscle is developed enough to cover both underlying palpebral laminae and Aditus Orbitae. TP appears as a thick cartilaginous structure that attaches LPS muscle. OS stands for the clear demarcation between pre-septal and post-septal fat. Extrinsic Ocular muscles, optic nerve, and supraorbital nerve were observed as well in their correct position. (Figure 7, 8, 9,10).

## Discussion

The embryologic studies of eyelids are of utmost rich in insights and compel, alongside fellow anatomy papers, to explain the cause-effect relationship implied in oculofacial pathology. As of this moment, scientists have yet to reach a unanimous opinion on how structures gather up in their definite frame and how every external factor affects the framework. Our work endorses previous data and stands to bring clarification to past debates in the postulated field. Moreover, our study raises new questions that can be tackled in future research.

Our attempt to simplify the developmental processes into 3 evolutionary stages is intended to ensure a broad understanding of the subject, thus making it possible to associate it with the clinical implications that may be encountered in pediatric ophthalmic pathology. Furthermore, we wish to establish a precedent for building the base of knowledge which could contribute at the earlier diagnosis of potential abnormalities.

The time of formation of eyelid folds comes in mismatch throughout many authors’ research, being thought of either as an earlier event (around week 6) by Tawfik et al.^10^ or a belated one (week 7) by Byun and Doxanas.^17,18^ We identified fused eyelids in specimens with a gestational age of approximately 7 weeks, while Byun et al. observed that this event is expected to happen by week 8^17^ – meaning that to our extent eyelid folds are existent from an earlier embryological moment. Yet, we were unable to state a precise time when folds become distinct macroscopically, since the youngest specimen was valued aging 6 weeks. We expect that assessing a higher number of young specimens to lead to more accurate results in the timeline of Stage A.

Nevertheless, eyelid morphogenesis respects the chronological sequence presented by previous studies.^10,14–16,18^ Moreover, there are several assumptions to raise with regards to the belief that during every developmental stage there should be at least one macroscopic or superficial feature that can be linked to the events happening microscopically or underneath the eyelid. Thus, we consider the groove that circumscribes Aditus Orbitae (noticed in stage A) to be the external indicator for the bone insertion point of the Orbital Septum. The transversal creases from stage A follow the Relaxed Skin Tension Lines of an adult eyelid.^25,26^ Moreover, the exophthalmic aspect of the eyeball and the manner in which it fades out are determined by viscerocranium growth and the development of the skin-underlying eyelid structures.

The changes suffered by the equatorial fusion line during stage B should be considered a macroscopic outcome of the keratin over-expression stated by Tawfik and Teraishi^10,27^ (by the time it resembles a protruding line), as well as an incipient presumed apoptosis process, postulated by Tawfik and Mohamed.^10,28^ This supports the belief that the peridermal cell layer is not mandatory in the eyelid closure process.^19,29,30^ We wish to unravel these premises in future research.

Regarding the dynamics of eyelid fusion and separation, both processes have been disputed from a molecular and morphologic point of view. It has often been argued whether the eyelid fusion begins either from the lateral canthus^10,18,31^ or from both lateral and nasal canthi.^10,32^ Acknowledging the fact that the fusion happens predominantly at a molecular level and both canthi are similar in aspect, we appreciate this discrepancy as rather irrelevant. However, bearing in mind that the fusion is initiated by the direct contact per se between the upper and lower eyelid and conducted by paracrine signaling,^21^ this is yet to be certified in humans as well. On the other hand, we have not encountered any Ankyloblepharon Filiforme Adnatum (AFA) cases that could support Sevel’s theory that eyelid separation takes place in a loose momentum throughout gestation rather than at a definite time.^10,16^ Even so, the low incidence of AFA,^33–35^ as well as the lack of fellow subjects included in our paper seem to support Tawfik, Doxanas, Anderson, and Byun’s postulates. We, however, tend to agree with Sevel, but histological slides from further case reports could explain both AFA pathogenesis and eyelid separation process, thus settling the dispute.

It is interesting to address the role of amniotic fluid in the development of the eyelid apparatus. Tawfik and Sevel stress the importance of eyelid fusion before the excretory system becomes fully functional, so that renal excretory products do not affect cell differentiation at the level of orbital structures.^10,16^ However, the hormones present in the amniotic fluid and their different concentrations according to gestational age, along with others, such as lecithin, glucose, and several proteins, could be linked to the eyelid opening in intrauterine life.^16,36–38^ Therefore, we consider the amniotic fluid as having a crucial role in the development of the eyelids; the timeline of the developmental stages of the eyelids must be per the global development of a fetus, and by this interplay, we come into recognition of how nature manages to keep embryology away from the primrose path – a phrase coined by Shakespeare in Hamlet, 1602, when Ophelia warned her brother to follow his own advice and not choose the easy route of sin over the tough and tedious path of virtue that leads to Heaven; also used to describe a delicate course of action among good and bad duality.

## Conclusions

Our main goal was to simplify the chronology of eyelid developmental events into 3 stages and to establish for each a distinct macroscopic feature. In stage A, an equatorial contour between eyelid folds is apparent commencing week 7 and marks the complete fusion of the eyelids. Surface projection of the superior Tarsal Plate and the appearance of the Superior Palpebral Sulcus take place in Stage B. Natural proportions and the approximation to the already known surface anatomy (of the face) are characteristic for Stage C. Employing macroscopical elements, we expect this study to stand as a basis for supposedly future guidelines in ultrasound diagnosis for the obstetricians, as well as to bring value in understanding ophthalmic-pediatric pathology and eyelid surgery.
